# Synchronization of passes in event and spatiotemporal soccer data

**DOI:** 10.1038/s41598-023-39616-2

**Published:** 2023-09-23

**Authors:** Henrik Biermann, Rumena Komitova, Dominik Raabe, Eric Müller-Budack, Ralph Ewerth, Daniel Memmert

**Affiliations:** 1 Institute of Exercise Training and Sport Informatics, German Sport University Cologne, Cologne, Germany; 2 L3S Research Center, Leibniz University Hannover, Hannover, Germany; 3 TIB—Leibniz Information Centre for Science and Technology, Hannover, Germany

**Keywords:** Applied mathematics, Scientific data

## Abstract

The majority of soccer analysis studies investigates specific scenarios through the implementation of computational techniques, which involve the examination of either spatiotemporal position data (movement of players and the ball on the pitch) or event data (relating to significant situations during a match). Yet, only a few applications perform a joint analysis of both data sources despite the various involved advantages emerging from such an approach. One possible reason for this is a non-systematic error in the event data, causing a temporal misalignment of the two data sources. To address this problem, we propose a solution that combines the SwiftEvent online algorithm (Gensler and Sick in Pattern Anal Appl 21:543–562, 2018) with a subsequent refinement step that corrects pass timestamps by exploiting the statistical properties of passes in the position data. We evaluate our proposed algorithm on ground-truth pass labels of four top-flight soccer matches from the 2014/15 season. Results show that the percentage of passes within half a second to ground truth increases from 14 to 70%, while our algorithm also detects localization errors (noise) in the position data. A comparison with other models shows that our algorithm is superior to baseline models and comparable to a deep learning pass detection method (while requiring significantly less data). Hence, our proposed lightweight framework offers a viable solution that enables groups facing limited access to (recent) data sources to effectively synchronize passes in the event and position data.

## Introduction

In recent years, soccer enjoyed a worldwide increase in popularity which was accompanied by an intensified interest of e.g. broadcasters and betting companies within the sport^2^. Documented by a large coincidental growth of the financial sector in professional soccer, not only the clubs (which can also be considered as companies, in some sense) but also large industrial groups are financially involved with soccer. An indication for the high importance of the sport for the global economy is given, for example, by the restart of various European top leagues during the 2020 pandemic^3^.

This development supported a recent growth in studies in the field of soccer analysis with a significant upsurge of data-driven approaches in the domains of computer science^4^. The mainly used data for these approaches can be divided into the two categories of position and event data, each providing a different perspective on the game.

Spatiotemporal *position data* are automatically captured by specific (camera) positioning systems^5, 6^, and describe the movement of the players and the ball on the pitch over time (predominantly in two dimensions). Here, the trajectories of the players and the ball are captured at a high rate (usually around 30 times per second) and provide good temporal resolution. Yet, please note that especially data from older camera systems is likely to contain parts with an incorrect spatial localization of, i.e., the ball position^7^, further described in Section “Post-processing”.

*Event data*, in contrast, describes the semantic flow of specific, human-defined events, such as passes, shots, and fouls, throughout the match and is typically captured by human annotators from various providers^8, 9^. This manual annotation of events is a very time-consuming process which has been reported to require three human annotators and two hours per match^9^. For this data type, the positions of the players surrounding an event are only recorded to some degree. This recently raised considerable criticism towards the event data where also the bias towards errors and the highly subjective characteristics of the events was discussed^10^.

In general, the two data types represent two varying perspectives on soccer analysis. On the one hand, the position data describes the match as a continuous stream of numbers representing a mathematical description of the sport. On the other hand, the event data comprise the perception of the respective annotator(s) within a context of predefined events representing the human understanding of soccer.

Due to the high complexity of soccer, a major challenge in the analysis is the interconnection of these perspectives to discover relevant information for the game. Approaches performing a coincidental examination of both data types are capable of projecting insights from the spatiotemporal data onto human-defined situations in the match. This involves benefits associated with a deeper understanding of the game and, possibly, the derivation of concrete recommendations for coaches and players. While these particular findings are highly valuable for the domain, to this date, only a few studies perform the simultaneous analysis of position and event data. Scoring probabilities for shots were estimated^7^ to provide additional information for viewers in a broadcasting application and the quality of passes was rated^11, 12^ to support (visual) match analysis. However concrete action recommendations from those studies can only be derived to a small degree.

Reasons for the lack of related research can be found in the and limited availability of synchronized data. It is common for event providers^8, 9^ to perform a manual annotation step for event data, leading a non-systematic temporal misalignment between the position and event data which was previously reported^7^ and is also empirically examined in this work. Accordingly, prerequisites for the simultaneous analysis of such data are either the manual alignment of the regarded events or the consideration of the synchronization error in the obtained results. To this end, automatic approaches that quantify and/or perform the synchronization of position and event data are highly valuable. For the research community, they present a method to sharpen the quality and quantity for analysis with existing data. For data providers, they extend the possibilities in the event data capturing process, e.g., by integrating additional post-processing and quality control steps.

A possibility to approach this issue is the automatic synthesis of event data, either from the position data itself or from another modality where synchronization with the position data is less complicated (e.g. video or audio data). The latter has been proposed^13^, however, here, passes are regarded as the duration in which the ball travels towards a teammate. As a consequence, the authors do not report an evaluation on an exact frame-wise annotation of passes which is yet required by a multitude of applications in this domain.

Vice versa, the detection of *atomic* pass events, defined as the moment in which the ball leaves the foot of the player, was also conducted^14^. The authors propose machine learning algorithms that are capable of detecting passes, along with other group activities, from either the video or the position data. The models are trained on a dataset of 74 matches of the 2018/2019 English Premier League season and achieve a decent detection performance with a slight advantage for the position data approach. However, the proposed machine learning methods require a large amount of data which is not practicable as the datasets are rarely publicly available^9^ and typically not easy to obtain. Specifically, in the domain of professional sports, the immense competitive pressure between different parties prevents openly accessible sources of position and event data.

Consequently, lightweight frameworks that require only small amounts of training data are preferred within the community. To this end, the synthesis of different types of soccer events has been proposed by Vidal et al.^15^. While their proposed algorithm is highly accurate, the authors state that the results are easily deterred by errors in the position data, restricting the applicability of their algorithm to older or less exact position data sources.

A feature-based approach that synchronizes *atomic* shot events from the event data with the position data by simultaneously integrating information from both data sources has been proposed by Bauer and Anzer^7^. The utilized feature-based approach is able to establish a synchronization for the examined shot events in the position and event data without requiring a large amount of data. However, the empirical evaluation is carried out on a comparably small number of 219 shot events. Moreover, their proposed algorithm includes additional spatial information (two-dimensional position of the shot event) from the event data which is not always accessible. Finally, the authors report issues with the quality of the position data which impedes the synchronization for certain passes, however, do not further detail on methods to account for this.

With the aim of advancing this approach, we design a framework for the synchronization of pass events (from event providers) with the position data. Therefore, we suggest a refinement step for imprecise event data that exploits a pass event detection in the position data. For this event detection we suggest a task-specific adjustment of the previously proposed SwiftEvent^1^ algorithm. Our algorithm aims for a frame-accurate annotation of pass events and also a reliable detection of examples with poor position data quality. We apply the synchronization algorithm to the pass event annotation of four matches of top-flight European soccer from the 2014/2015 season. On this dataset, we find that the refinement significantly improves the precision of the annotation and show that the detected examples involve a significant amount of noise.

The obtained results are comparable to a state-of-the-art machine learning scenario^14^ while our utilized dataset is substantially smaller in size and captured by less recent tracking systems. Thus, we find that this work provides a valuable contribution to the state of the art as it realizes the pass synchronization for position and event data with low requirements regarding data quantity. As the presented method furthermore accounts for problems with the position data quality, it is particular valuable for analysis on less recent data. This benefits groups that encounter the issue of low availability of (especially recent) soccer position and event data as it allows for performing a combined analysis.

The remainder of this work is structured as follows: Section “Related work” outlines related work in soccer and time-series analysis. Section “Pass event synchronization using time-series analysis” details on the synchronization of pass events and Section “Evaluation” illustrates the evaluation of the algorithm. Finally, Section “Conclusions” draws conclusions regarding the achieved improvement and discuss implications for future work.

## Related work

In the general field of *time-series analysis*, we observe four different categories. Namely *forecasting*^16–18^, *event detection*^19–21^, *clustering*^22–24^ and *classification*^25–27^. In this work, we design an algorithm to perform a synchronization of two sources of soccer time-series data by regarding the detection of pass events in the position data.

Time-series event detection algorithms relate to data-mining techniques and, thus, focus on detecting specific patterns in the time-series. A discrimination of approaches in this topic is made by examining the utilized methods which can be divided into classical statistical and novel machine learning approaches^28^. A comparison of these methods in the field of time-series forecasting demonstrated that statistical methods outperform machine learning approaches for the majority of the time^28^. Although statistical methods in the field of event detection are well-examined (cf. change point detection)^29^, the similarities to other pattern recognition tasks promoted various studies addressing the application of supervised and unsupervised machine learning algorithms^30^. Accordingly, the majority of recent approaches in time-series event detection are based on machine learning techniques while only a few studies investigate the performance of statistical methods.

The specific task of *soccer event detection* has been successfully implemented by various approaches performing statistical and machine learning on video data^31–40^, audio data^31, 41, 42^ and data gathered from social networks^43^. The focus of these approaches lies on automatically creating a summary of the match, which is a function of high interest for viewer-based applications, e.g., for the creation of highlight videos. Consequently, the research in recent years mainly addressed the detection of infrequently occurring important events where a precise determination of an exact frame is less important.

In detail, the detection of goals as the most important event in soccer was targeted by multiple studies. A heuristic method focusing on the intensity of video and audio data was introduced^31^. Two independent event detection algorithms on video data were utilized to perform the real-time detection of scores and near misses^34^. Furthermore, the detection of probable scoring opportunities from video data was proposed^39^. Additionally, a variety of studies performs the summarization of the match by detection of more detailed events beneath scores. The application of complex machine learning techniques to detect goals, shots, corner kicks, and cards was proposed^33^. Similar studies also address the recognition of replays in broadcast videos to accordingly detect different event types using support vector machines^32^, convolutional neural networks^35^, multiple instance learning^38, 41^, trajectory-based deep convolutional descriptors^36^, or a combination of support vector machines and neural networks^40^. The modality of auditory data was also used in various approaches^31, 41, 42^ where, for instance, the sentiment of the commentators was analyzed along situation-specific sounds to detect highlights. Another modality has been proposed by Van Oorschot et al.^43^, who scraped posts from social networks which comment on the match to recognize important events.

Contrarily, only a few studies lay the focus on the detection of events occurring with high frequencies (passes, shots, tacklings). An approach similar to the previously presented studies applied Bayesian networks to broadcast videos^37^ and was able to further detect non-highlight events. The automatic detection of ball possession of individual players based on video data was implemented by using object detection methods and deep learning^44^. A similar study applied long short-term neural networks on video data to automatically detect time spans where a pass is played^13^. In contrast, a frame-accurate annotation of *atomic* pass events without a duration was conducted by a machine learning action approach using self-attention on both, video and position data^14^. The latter study detected shots, receptions, and passes from the respective data without additional information about the individual player or event. However, since this information can be obtained from, e.g. the event data, an application that utilized this information to synchronize shots in the event data with the position data was already proposed^7^. A computationally less demanding approach for event detection has been proposed by Vidal et al.^15^, however, their algorithm requires high-quality, exact position data which restricts the applicability in cases where this data is not available.

In this work, we expand the previously presented study to the synchronization of passes. Therefore, we initially perform a general pass event detection using the SwiftEvent algorithm^1^ by conducting a feature extraction and probabilistic classification. Subsequently, we apply the detection algorithm to refine the imprecise pass annotation from the event data. We evaluate the performance of the algorithm by performing a 4-fold cross validation of our dataset and show that our proposed methods strongly improve the degree of synchronization for the passes in event and position data. In contrast to previously proposed machine learning algorithms, our approach requires a very small amount of data and is able to detect localization errors in the position data. Thus, this work addresses research groups with limited availability of recent position data who aim to perform a coincidental analysis of passes in the spatiotemporal position and event data.

## Pass event synchronization using time-series analysis

In this section, we describe the proposed methods to improve the synchronization of pass events in soccer games using position and event data. First, we define the problem and present our input data. Second, we discuss relevant information to detect passes in the position data. Regarding the synchronization of shots, a previous algorithm proposed using player-ball distance and ball acceleration^7, 45^. Consequently, we adopt this procedure for our approach and present the computation of these signals from the position data in Section “Computation of player-ball distance and ball acceleration time-series”, the segmentation into *time-series windows* in Section “Segmentation of player-ball distance and ball acceleration time-series”, and post-processing steps in Section “Post-processing”.

Concerning the methods for the detection of passes, we need to deal with the novelty of the apparent task and the lack of research in this specific domain. As pointed out in Section “Introduction”, the highly competitive nature of professional soccer prohibits the majority of applications to assess large datasets. Therefore, we want to utilize different methods than previously proposed large-scale machine learning algorithms^14^ and decide on a lightweight framework.

**Figure 1:**
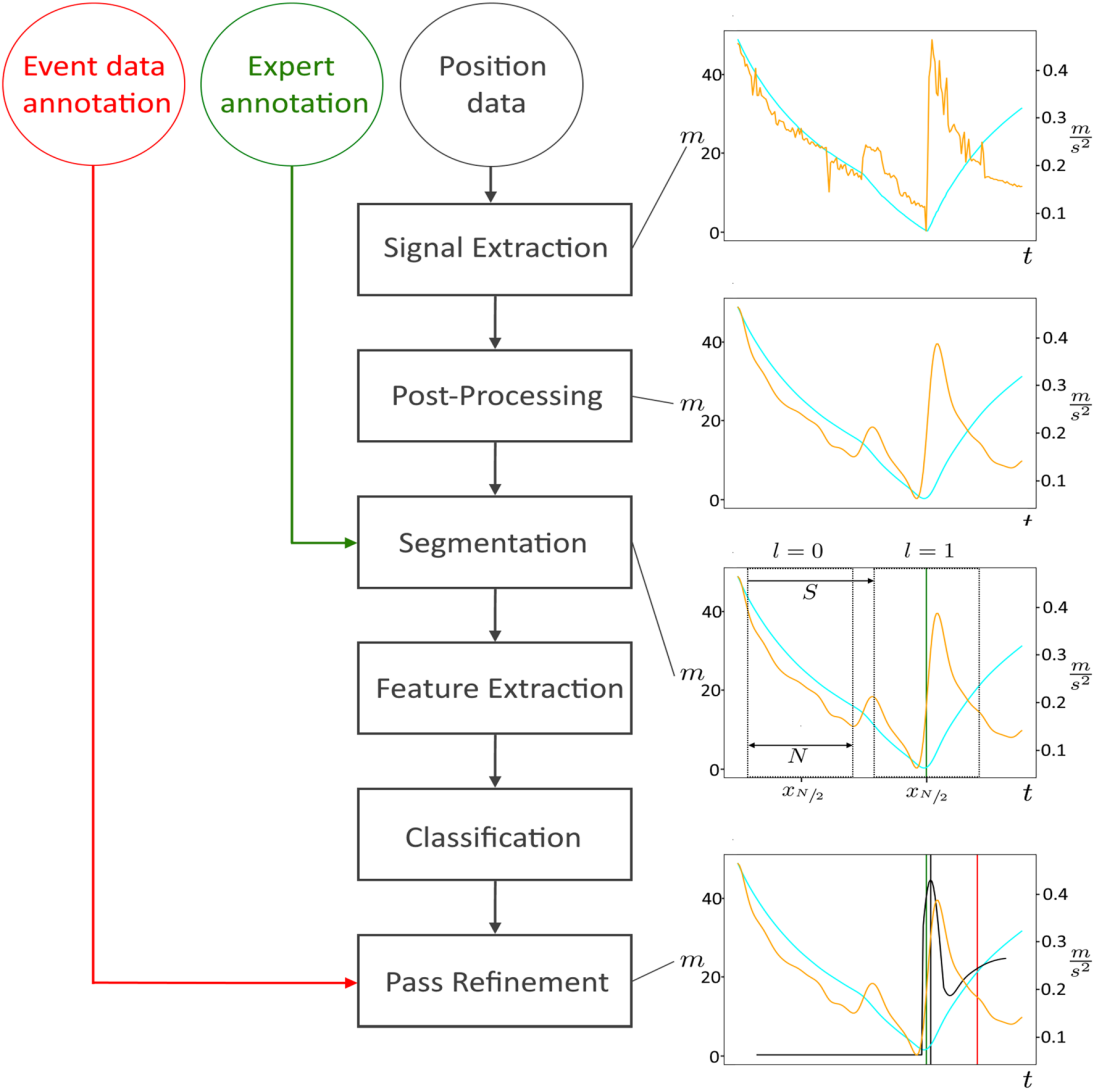
The diagram illustrates the Workflow of the proposed algorithm. On the left, the input data and the involvement in the respective steps of the algorithm is displayed. On the right, an exemplary sequence for player-ball distance (cyan curve, in *m*) and ball acceleration (orange curve, in $\frac{m}{s^2}$) for different steps of the algorithm are presented. The bottom two plots on the right part include the respective *expert pass label* (green line), the *imprecise pass label* (red line), and the *refined pass label* (black line, quantitative) which is computed from the algorithm’s *pass event probabilities* $P(\varvec{f}_w^r)$ (black curve).

Regarding the specific design of features, we can not rely on a feature space that has already been evaluated. A previous approach^7^ used features originating from an additional spatial annotation in the event data, which, however, is not always included in the event data. Thus, we can not simply adopt the utilized feature space. To this end, we perform a thorough investigation of a broad number of features, listed in the Appendix. We describe the feature extraction process in Section “Feature extraction” and illustrate the estimation of parameters from the features Section “Pass event detection” to construct a classifier performing pass event detection. We detail on the application of this classifier for refining pass annotations in the event data in Section “Pass event refinement and outlier detection”. The entire workflow is further visualized in Fig. 1.

### Problem definition

Given the position data, the event annotation data (consisting of imprecise temporal annotations for passes in the match from a data provider), and the expert annotations (consisting of precise temporal annotations of passes in the match), our objective is (1) to establish a general pass event detection from the expert annotation and position data and (2) to apply it to the imprecise event annotation for passes to estimate the exact time when the ball left the foot. We repeat this procedure for all passes in the match to ultimately improve the degree of synchronization between these data sources.

*Positional data:* The spatiotemporal data is automatically captured by specific camera systems^5^ or positioning systems^6^ and contains trajectories of the players and the ball. We regard it as temporally exact and define it as the synchronization target for the refinement of the event data. For a specific match, we obtain positions in two coordinates for all *R* players on the pitch and the ball captured with *sampling frequency* of $f_s = 25~\text {Hz}$. We define the position of an individual player $r = 1,\ldots , R$ at frame $k = 1, \dots , K$ as a 2-tuple (an ordered pair) $m^{r}(k) = (m_x^r(k), m_y^r(k))$. Here, *K* refers to the total number of samples during the match, while $m_x^r(k)$ and $m_y^r(k)$ denote the current *x*- and *y*-coordinate of player *r* at frame *k*, respectively. Furthermore, we define the specific *player position vector* $\textbf{m}^{r} = \left[ m^{r}(1), ..., m^{r}(K)\right] ^\text {T} \in \mathbb {R}^{K}$ with the *K* player position tupels over the course of the match as elements. Analogously, we introduce the *ball position vector* $\textbf{b} = \left[ b(1), ..., b(K)\right] ^\text {T} \in \mathbb {R}^{K}$ with elements $b(k) = (b_{x}(k), b_{y}(k))$, where $b_{x}(k)$ denotes the *x*-coordinate and $b_{y}(k)$ denotes the *y*-coordinate of the ball. Finally, we combine the individual player positions for players $r = 1, \dots , R$ in the aggregated *player positions matrix* $\textbf{M} = [\textbf{m}^1, ..., \textbf{m}^{R}] \in \mathbb {R}^{K \times R}$. The entire spatiotemporal position data of the match is thus captured in the matrix $\textbf{M}$ and the vector $\textbf{b}$. We account for substitutions by appending the player-ball distance of the in-sub to the distances of the respective out-sub, and for red cards or injuries, by appending zeros to the suspended player’s column in $\textbf{M}$.

*Event Data and Error Analysis:* Event data for soccer matches can be obtained from various providers, (e.g.^8, 9^). It is typically captured by human annotators and its annotation can therefore be temporally imprecise. Here, two different types of errors are encountered: (1) A *systematic* error in the absolute timestamps of the positions and the event data, and (2) a *non-systematic* error which largely varies along different passes^7^. While the compensation of the latter is the primary task of our approach, we also need to account for the systematic error. This error can be caused by various circumstances, e.g. the human event data annotator watching the match from a broadcast video by either terrestrial transmission or satellite transmission with respective transmission delays. We compensate the systematic error as previously proposed^7^ by regarding *relative timestamps* in both data sources which are respectively computed as the temporal difference of the timestamp to the kickoff. This way, we examine the pass annotation for four matches in professional European soccer for which we obtain video and position data along with the event data. From the latter, we gather the *relative timestamps* as well as information about the passing player. We group all $N_u$ pass annotations $l^u_1,\ldots , l^u_{N_u}$ during a match and denote them as *imprecise pass labels* $L^u = \{l^u_1,\ldots , l^u_{N_u}\}$. The information about timestamp and player is then stored as 2-tuple $l^u_i = (k^u_i, r^u_i)$ where $k^u_i \in \{1, \dots , K\}$ is the timestamp of the pass and $r^u_i \in \{1, \dots , R\}$ is the passing player.

**Figure 2:**
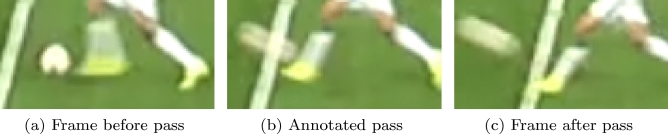
Visualization of a representative pass in the video data. Precise *expert pass labels* were acquired by domain experts that have specified the exact frame of each pass in a football match.

**Figure 3:**
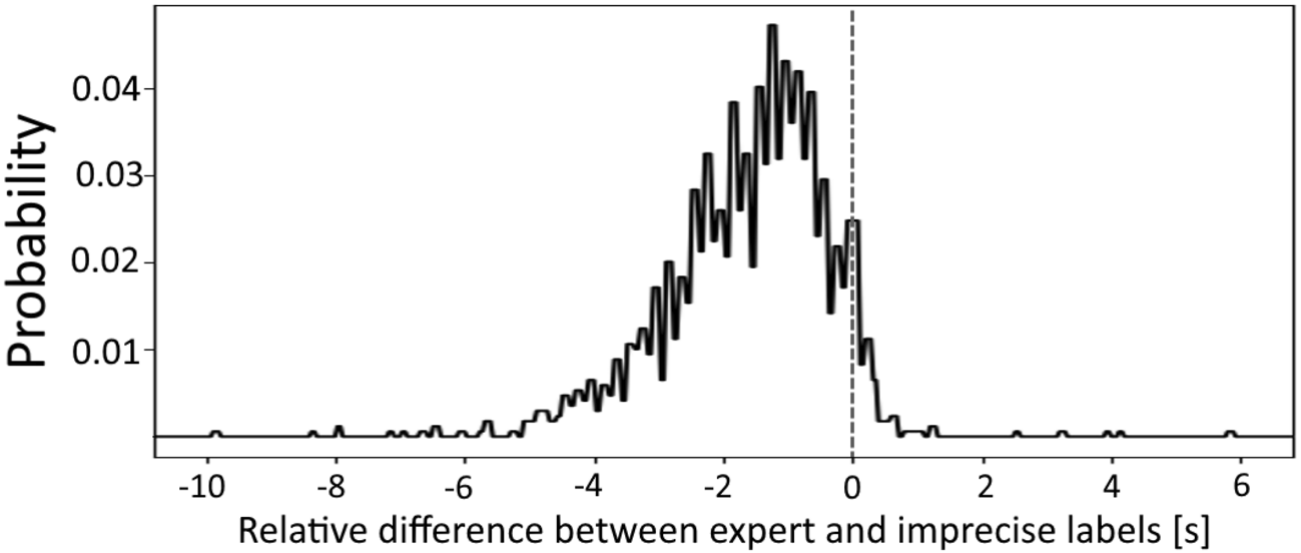
Sampled temporal difference of the *imprecise pass labels* compared to the *expert pass labels* for valid sequences (see Section “Dataset”) from all matches in our dataset within a cut interval for better visualization.

*Annotation of Precise Expert Pass Labels:* To further investigate the non-systematic error, we carefully acquire precise pass labels for the four matches through annotations of a domain expert. The expert determines the exact point in time where the ball left the foot of the respective passing player by analyzing the video frame by frame, as illustrated in Fig. 2. For this purpose, we implement a custom application that realizes the projection of the annotated passes from the video to the position data. We refer to the total $N_p$ manual annotations as *expert pass labels* $L^p = \{l^p_1, ..., l^p_{N_{p}}\}$ with $l^p_i = (k^p_i, r^p_i)$ analogously to above. This way, we are able to sample the non-systematic error between the *expert pass labels* and the *imprecise pass labels*. The systematic error is exemplary displayed in Fig. 3 and utilized in Section “Comparison to baselines and state of the art” for the design of a *statistical baseline* method which addresses the systematic error of delayed pass annotations in the original event data.

### Computation of player-ball distance and ball acceleration time-series

As already introduced in Section “Problem definition”, we describe the position data of a match in terms of the *player positions matrix* $\textbf{M} \in \mathbb {R}^{K\times R}$ and *ball position vector* $\textbf{b}\in \mathbb {R}^{K}$, where the single entries $m^{r}(k)$ and *b*(*k*) represent 2-tuples with the two-dimensional field position of player $r = 1, \dots , R$ and the ball, respectively. To detect shots in the position data using player-ball distance and ball acceleration, a previous approach has been proposed^7^. Thus, we aim to exploit these quantities in the detection of passes in the position data and present the computation of player-ball distance and ball acceleration time-series from the position data. We compute the distance of player *r* to the ball at frame $k=1, \dots , K$, i.e. $d^r(k) = \Vert m^r(k) - b(k)\Vert$ with $\Vert \cdot \Vert$ being the Euclidean norm. Furthermore, we introduce the ball acceleration *a*(*k*) that approximates the current real ball acceleration at frame *k*. To compute *a*(*k*), we take the first derivative of the ball velocity in one step as $a(k) = v^\prime (k) = b(k+1) - 2b(k) + b(k-1)$, where the interval between adjacent points is one.

### Segmentation of player-ball distance and ball acceleration time-series

The previous section presented the computation of player-ball distance and ball acceleration time-series from the position data of a match. In this section, we discuss the segmentation of the previously computed time-series. We therefore choose a sliding window approach to separate time-series into *windows* (with fixed *window length* *N*) while also allowing for an overlap between *windows* (according to the chosen *window shift* *S*).

The goal of this is to divide the information time-series into smaller parts describing short periods of the match. To achieve this, we individually segment the time-series to obtain *player-ball distance windows* and *ball acceleration windows*. If we combine these we obtain *time-series windows* which can be collected for a player during a match to obtain the player specific *time-series window matrix*. As this matrix contains the previously determined decisive factors for the detection of passes (see Section “Computation of player-ball distance and ball acceleration time-series”), we can apply it for training a classifier that processes individual segments and makes statements about the probability of a pass in the segment. However, this requires the assignment of labels for individual *time-series windows*. Therefore, we regard the expert event annotation for the match, which we obtain additional to the position data. From this source we collect the *expert pass labels* $L^p$ (see Section “Problem definition”).

**Figure 4:**
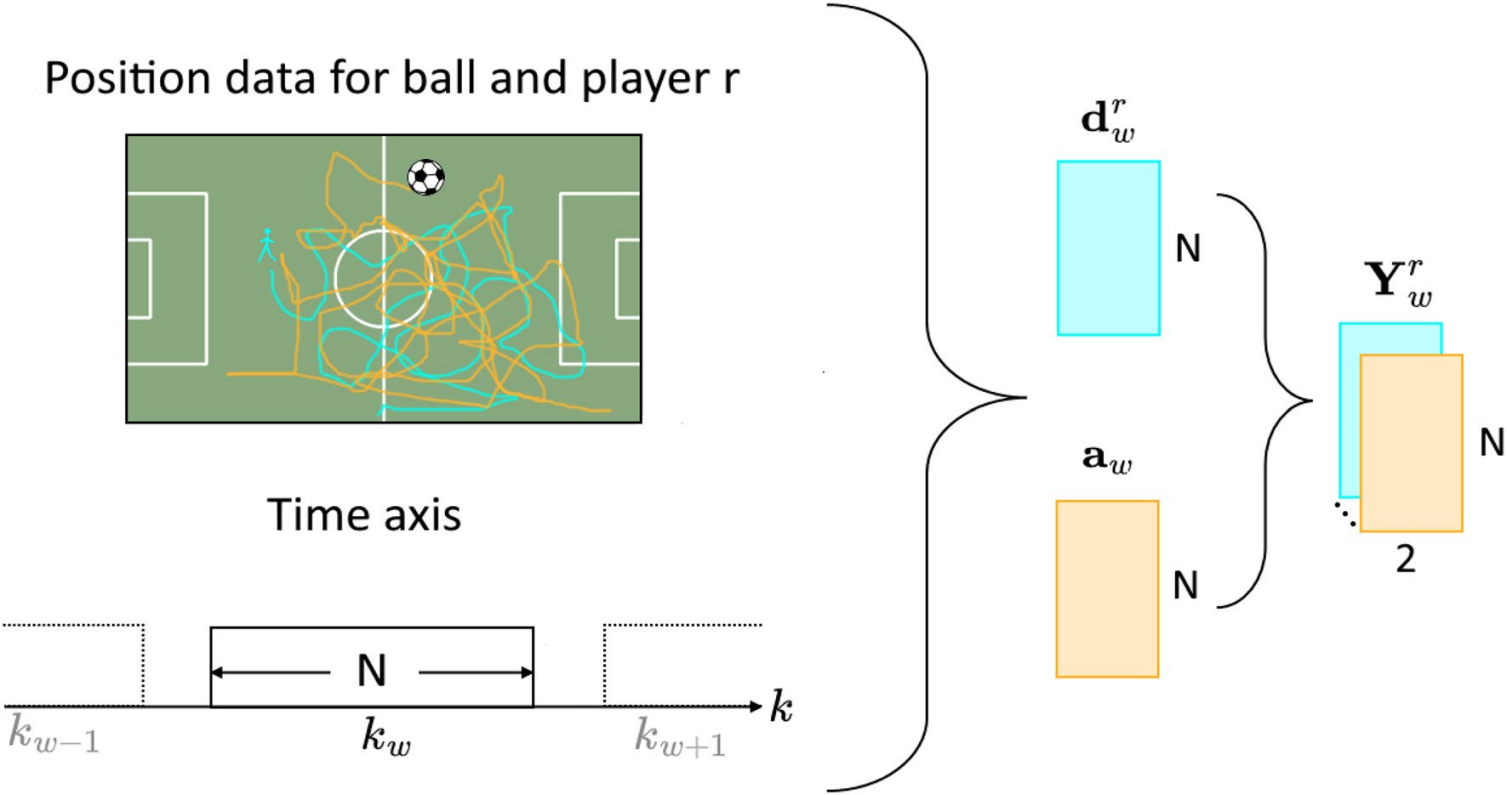
Visualization of the construction of a single *time-series window* $\textbf{Y}^r_w$ for a single player *r* from the player-ball-distance (cyan) and ball acceleration (orange) windows $\textbf{d}^r_w$ and $\textbf{a}_r$ which are computed from the position data within the window $k_w$ with window length *N*.

Given are the frames *k*, $k=1, \dots , K$, representing the time axis of the whole match. Now segment this time axis into *windows* $k_w= [k_w^1, \dots , k_w^N]$ with the *window length* *N* and *window shift* *S*, where the individual *window* samples $k_w^n$, $n = 1, \dots , N$, are computed by $k_{w}^{n} = k_{wS + N + n}$. Using this definition, we divide the ball acceleration time-series into *ball acceleration windows* $\textbf{a}_w \in \mathbb {R}^{N}$ with $w = 1, \dots , W$ being the number of *windows* within a match. Analogously, we segment the player-ball distance into player specific *player-ball distance windows* $\textbf{d}^{r}_{w} \in \mathbb {R}^{N}$ with $r = 1, \dots , R$. Finally, aggregate all relevant information for player *r* about player-ball distance and ball acceleration into player specific *time-series window matrix* $\mathbf {Y^r} \in \mathbb {R}^{W \times (N \times 2)}$ with *N* individual *time-series windows* $\textbf{Y}^{r}_{w} \in \mathbb {R}^{N \times 2}$ given as $\textbf{Y}^{r}_{w} = [\textbf{d}^{r}_{w}, \textbf{a}_w ]$ containing the stacked *player-ball distance* and *ball acceleration windows*. The complete segmentation process is illustrated in Fig. 4.

In the following step, we assign negative and positive *window labels* $l^{r}_{w} \in \{0, 1\}$, $w=1, \dots , W$, to each player $r = 1, \dots , R$ a specific *time-series window matrix* $\mathbf {Y^r}$. Regarding the *window center* $k_w^{n_c}$ with $n_c = \lfloor \frac{N}{2} \rfloor$, where *N* is the *window length*, we assign a positive *window label* $l^r_w = 1$ if and only if an *expert pass label* $l^p_i = (k^p_i, r^p_i)$ of passing player $r^p_i = r$ exists that has a timestamp at the *window center*, i.e. it holds $k^p_i = k_w^{n_c}$. Otherwise, we assign a negative *window label* $l^{r}_{w} = 0$. Please note that this process might require a tolerance (analogous to the event detection zone^1^) around the *window center* $k_w^{n_c}$ if the *window shift* *S* is greater than $\frac{1}{f_s}$, with $f_s$ denoting the *sampling frequency* (see Section “Problem definition”).

Ultimately, we emphasize that the procedure described in this section could easily be extended to an online approach which is technically realized by a fixed *time-series window* where the last data point of the *window* is iteratively replaced by a new data point. Accordingly, we choose the *window shift* of $S=\frac{1}{f_s}$, which corresponds to updating a fixed *time-series window* by a single frame at each iteration.

### Post-processing

The automatically captured spatiotemporal position data from video tracking systems, although temporally precise, can show a partly unstable and inaccurate behavior, which has already been reported^7^ and especially affects the ball position data. For example, the ball could be invisible to (some cameras in) the multi-camera system due to occlusions, e.g., with the crowd when a lofted pass with high trajectory was performed. In such cases, the ball position is often assigned to a nearby player position until it again becomes visible to the tracking systems. However, this process of visually retrieving the ball can consume some time even after the ball technically became visible again for the video tracking system.

As a consequence of this kind of incorrect localization, individual values of player-ball distance and ball acceleration are erroneous. We partially compensate this by our post-processing step via a low-pass filter with cutoff-frequency $f_c$. If the cutoff is chosen accordingly, the low-pass is able to smooth the time-series by excluding high frequencies from the signal (see example in Fig. 1) while preserving its envelope. Since these frequencies are likely to originate from undesired artifacts (or noise) in the tracking process, this procedure can enhance signal quality. Admittedly, this does not apply to long periods of incorrect localization. Thus, we address the detection of passes with faulty position data in Section “Pass event refinement and outlier detection”.

### Feature extraction

Since, in general, the size of the previously presented *time-series window matrix* $\mathbf {Y^r}$ depends on the segmentation parameters *N* and *S* (see Section “Segmentation of player-ball distance and ball acceleration time-series”), it can develop to be very large (c.f.  “Dataset” Section). Thus considering memory and computing time limitations, we regard it as favorable to reduce the amount of data by extracting descriptive features from the individual *time-series windows* $\textbf{Y}^{r}_{w}$.

**Figure 5:**
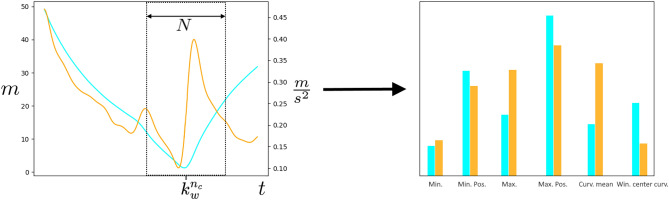
*Left*: Exemplary sequence for a single player-ball distance (cyan) and ball acceleration (orange). *Right:* Separate extraction of selected features representation from the segment.

Concerning the specific design of the features, we need to deal with the novelty of the apparent task and the lack of research in this specific domain. As pointed out at the beginning of this section, we can not simply adopt the utilized feature space. Therefore, we separately investigated a broad number of different suitable features for the characterization of player-ball distance and ball acceleration. Among other descriptive features, we choose the minimum and maximum values and their respective position within a *time-series window*, the value at the *window center*, the curvature mean, and the curvature at the *window center*. Here, the curvature is approximated as the instantaneous frame difference, e.g., for a time-series *y*(*k*) at sample *k* as $[y(k+1) - y(k-1)]/2$. Moreover, we separately conduct a polynomial approximation for player-ball distance and ball acceleration within the *time-series windows* and use the obtained weights for this approximation as a feature. We illustrate the extraction process for a representative choice of features in Fig. 5 and refer to the Appendix for a complete list of examined features and a detailed explanation of the polynomial approximation procedure.

The completed extraction of individual descriptive features from the *time-series windows* $\textbf{Y}^{r}_{w}$ allows the introduction of a feature representation vector $\varvec{f}^r_w \in \mathbb {R}^D$, which respectively comprises a combination of *D* descriptive features for either player-ball distance or ball acceleration (see Fig. 5, right). We performed extensive evaluation of various combinations of features (listed in the Appendix) and decided on three distinct feature configurations (see Table 1) that achieved the highest performance in explorative experiments while being manageable in their complexity. Another finding from these experiments is that such lightweight feature configurations perform similar to more complex configurations (i.e. using all listed features from the Appendix). This complies with the SwiftEvent algorithm that works on low-dimensional feature spaces to enable a computation in real time. This is an important aspect of our algorithm regarding the real world applicability for different tasks (see Section “Introduction”). For the sake of conciseness, we thus decide for Section “Evaluation4” to only to report detailed experimental results for the three configurations from Table 1.

**Table 1:** Overview of feature configurations.

	Player-ball distance features	Ball acceleration features	*D*
FR 1	Curvature mean, Minimum position	–	2
FR 2	3 Polynomial weights, Minimum, Minimum position	Curvature mean, Maximum position	7
FR 3	6 Polynomial weights, Minimum, Minimum position, Curvature mean, *Window center* curvature	Curvature mean, Maximum position	12

### Pass event detection

We design an event detection algorithm for passes as a binary classification problem which unfolds based on the extracted feature representations (see Section “Feature extraction”) of the individual *time-series windows* (see Section “Segmentation of player-ball distance and ball acceleration time-series”).

Due to the absence of large-scale datasets labeled for precise pass events^9^, we want to perform the pass event detection with a lightweight probabilistic framework that requires only a small amount of training data. Moreover, to ensure applicability our approach also requires to run in real time. These requirements are fulfilled by the SwiftEvent algorithm that has been proposed for the feature-based supervised event detection in time-series^1^. Thus, we adopt the general workflow of the algorithm, however, while refining it for the given pass event detection problem.

Our player-ball distance and ball acceleration time-series are given within *windows* $k_w$ with $w=1, \dots W$ and players $r=1, \dots R$. The occurrence of events is indicated by *window labels* $l^r_w$ (see Section “Segmentation of player-ball distance and ball acceleration time-series”) and we extract feature representations $\varvec{f}^r_w \in \mathbb {R}^D$. We follow the SwiftEvent algorithm^1^ which assumes the features are normally distributed random Gaussian variables, i.e. $\varvec{f}^r_w\sim \mathcal {N}(\varvec{f}|\varvec{\mu }, \varvec{\varSigma })$^1^ and compute the suggested Mahanalobis distances $\varvec{\varDelta }_{\varvec{\varSigma }_{0}}(\varvec{f}^r_w)$ and $\varvec{\varDelta }_{\varvec{\varSigma }_{1}}(\varvec{f}^r_w)$ to the centers of the distributions for the two *window label* classes $l^r_w = 0$ and $l^r_w = 1$. Similar to SwiftEvent, these distances serve as criteria in the detection of an event within *window* $k_w$.

However, opposing to Gensler and Sick^1^ we do not evaluate learned thresholds. In contrast, we propose a novel method to compute probabilities for the present pass event detection problem where we expect that the number of labels $l^r_w = 0$ in the binary classification task significantly exceeds the number of labels $l^r_w = 0$. Accordingly, we define the *pass event probability* $P(\varvec{f}_w^r)$ for $w=1,\ldots , W$ as

1 $$\begin{aligned} P(\varvec{f}_w^r) = {\left\{ \begin{array}{ll} \overline{\mathbb {P}}(\varvec{f}^r_w), &{} \text {if}\quad \varvec{\varDelta }_{\varvec{\varSigma }_1}(\varvec{f}^r_w) \le \varvec{\varDelta }_{\varvec{\varSigma }_0}(\varvec{f}^r_w) \\ 0 &{} \text {otherwise}, \end{array}\right. } \end{aligned}$$

such that the Mahanalobis distance to the *window label* class $l^r_w = 1$ needs to be lower than the distance to the *window label* class $l^r_w = 0$ before a non-zero prediction is made. Moreover, to receive probability values in the range [0, 1] we define the normalized probability $\overline{\mathbb {P}}(\varvec{f}^r_{w})$ as

$$\begin{aligned} \overline{\mathbb {P}}(\varvec{f}^r_w) = \frac{\mathbb {P}(\varvec{f}^r_w)}{\mathbb {P}(\varvec{\mu }_{1})} \end{aligned}$$

with $\mathbb {P}(\varvec{f}^r_w)$ being the probability of the normally distributed random variable divided by the probability at the distribution mean $\varvec{\mu }_{1}$, computed from all feature representations with *window label* class $l^{r}_{w}=1$ as

$$\begin{aligned} \varvec{\mu }_{1} = \frac{1}{W_{1}} \sum ^{R}_{r=1}\sum ^{W_{i=1}}\varvec{f}^r_{i}. \end{aligned}$$

Here, $W_1$ represent the number of *windows* with a positive *window label* $l^r_w = 1$ for player *r*.

### Pass event refinement and outlier detection

To refine the existing *imprecise pass labels* from the event data source, we utilize the previously discussed general pass event detection (see Section “Pass event detection”) to implement an informed maximum a posteriori (MAP) estimation.

Thus, we inspect the *pass event probabilities* in the neighbourhood of a given individual *imprecise pass label* $l^u_i = (k^u_i, r^u_i)$ with $r^u_i = r$ and search the feature representation $\varvec{f}^r_w$ with the highest *pass event probability* $P(\varvec{f}^r_w)$ (described in  “Introduction” Section) within a generally defined *search interval*. This interval is chosen by closely inspecting the sampled temporal error of the *imprecise pass labels* (see Fig. 3) such that the majority of the total errors lie within.

We initialize the refinement process by searching the *time-series window* $\textbf{Y}^{r}_{w_u}$ at *window* $k_{w_u}$ with underlying *window center* $k^{n_c}_{w}$ being closest to $k^u_i$. We then examine the set of *windows* $K^s_i = \{k_{w_v}, \dots , k_{w_o}\}$, where $k^{n_c}_u$ and $k^{n_c}_o$ respectively are the smallest and largest *window centers* that still lie within the search interval.

Subsequently, we apply our event detection algorithm to the the *time-series window* $\textbf{Y}^{r}_{w}$ to compute *pass event probabilities* $P(\varvec{f}_w^r)$ for all feature representations $\varvec{f}_w^r$ that originate from *windows* $k_w \in K^s_i$.

Thereupon, we decide on the *refined time-series window* $\textbf{Y}^{r}_{w, \text {opt}}$ according to the maximum *pass event probability* $P(\varvec{f}^r_{w, \text {opt}})$ with

2 $$\begin{aligned} P(\varvec{f}^r_{w, \text {opt}}) \ge P(\varvec{f}^r_{w}) \quad \text {for all} \quad w = w_v, \dots , w_o . \end{aligned}$$

In consequence, we regard the *window center* $k_{w, \text {opt}}^{n_c}$ of the *refined time-series window* $\textbf{Y}^{r}_{w,\text {opt}}$ as the frame-wise exact *refined pass label*.

Finally, we utilize the previously computed maximum *pass event probability* $P(\varvec{f}^r_{w, \text {opt}})$ as in (2) to approach the detection of outliers. We refer to an *expert pass label* (annotated from the video data) as an outlier if its underlying position data contains a large amount of noise. This is caused, for instance, by the previously mentioned poor localization of the ball (see Section “Post-processing”) and is manifested by non-realistic behavior of player-ball distance and ball acceleration at the *expert pass label*, e.g. a large ball distance of the passing player (see Fig. 8) and low ball acceleration (see Fig. 2). Since such unrealistic behavior also correlates with a low maximum *pass event probability* $P(\varvec{f}^r_{w, \text {opt}})$, we are able to detect outliers by regarding $P(\varvec{f}^r_{w, \text {opt}})$ as a confidence score and introducing a *detection threshold* $\tau$ in the refinement process. If this threshold is not exceeded by $P(\varvec{f}^r_{w, \text {opt}})$, no improvement of the *imprecise pass label* is done and it is recognized as an outlier. This way, the algorithm is capable to detect localization errors in the underlying position data at the *expert pass labels* according to specific requirements of particular applications.

## Evaluation

In this section, we outline different experiments to evaluate the performance of our proposed algorithm and the impact of its components. Therefore, we initially describe the utilized dataset and introduce a prerequisite for computing errors between *imprecise pass labels* and *expert pass labels*. We then discuss metrics to assess the pass refinement and noise removal. Based on these metrics, we conduct a selection of optimal parameters in the algorithm and, finally, compare the optimal configurations with three baselines.

### Dataset

**Figure 6:**
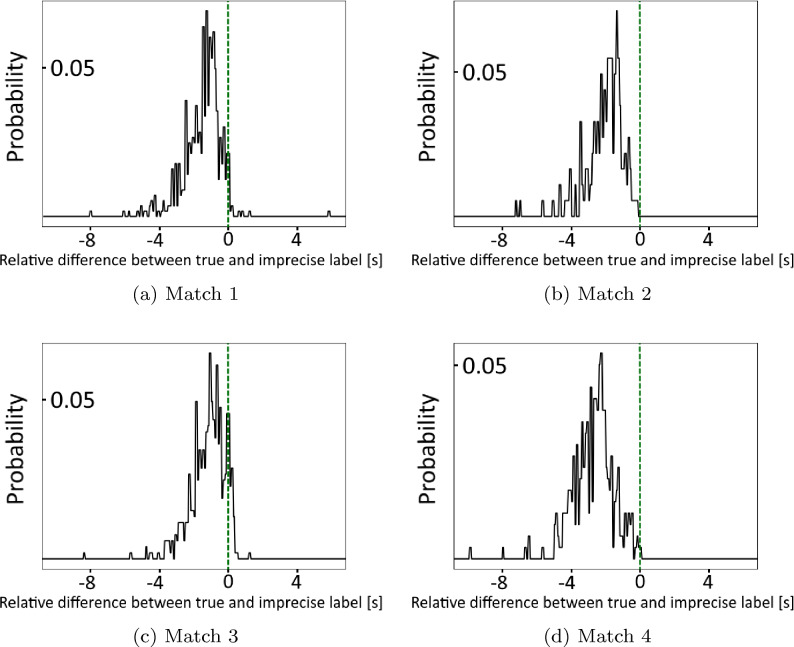
Schematic display of temporal difference of the *imprecise pass labels* compared to the *expert pass labels* for valid sequences (see Section “Dataset”) within the same cut interval as in Fig. 3, separately presented for the four matches in our dataset.

Our dataset comprises four matches from European top-flight soccer from the 2014/15 season with a total frame count of 280, 187 positional data points. We present the errors of the event data annotation for the four matches in Fig. 6. In total, $N_u = {2404}$ *imprecise pass labels* and $N_p = {2552}$ annotated *expert pass labels* (see Section “Problem definition”) were obtained for those matches. As a result, 2552 *time-series windows* $\textbf{Y}^{r}_{w}$ labeled for a valid pass event ($l_{r, w}=1$) were extracted. The remaining *time-series windows* are labeled with a negative *window label*, i.e. $l^{r}_{w}=0$.

To evaluate the algorithm, we perform four-fold cross-validation by splitting the dataset into training data comprising three matches and test data containing the remaining match.

#### Training

For training, we extract a total of $W_\text {train}$ *time-series windows* $\textbf{Y}^{r}_{w}$ with *window labels* $l^{r}_{w}$ (see Sections “Computation of player-ball distance and ball acceleration time-series”–“Post-processing”) from three of the four matches. From the *time-series windows* we compute feature representations $\varvec{f}^r_w$, $w=1,\ldots , W_\text {train}$, as described in Section “Feature extraction”. Training is completed by utilizing the representations and their assigned *window labels* for estimating the parameters of the probabilistic distributions in order to construct the *naïve Bayes classifier* for the pass event detection.

#### Testing

As previously mentioned the line between a pass and another action (i.e. a shot or a tackle) is not well established. Therefore, it is often difficult to decide on a definitive number of ground truth passes in a game. Analogously, the number of *imprecise pass labels* and *expert pass labels* in our dataset differs (see  “Dataset” Section). While this is not further problematic for the training of the algorithm, it poses a problem for the evaluation step as detection errors can not be computed by simply subtracting the passes according to the order of appearance. The straightforward way to approach this issue is to assign each detected pass its nearest expert pass label (Nearest Neighbour Matching) which was previously applied^14^. However, it has been argued that this assignment can introduce a positive bias to the evaluation results that originates from possible many-to-one mappings^46^. Due to the fact that our proposed algorithm provides a refinement step for existing pass event annotations, a more suitable way to calculate meaningful error metrics is given by one-to-one mappings between expert and imprecise pass labels. Thereupon, we use the previously introduced^46^ *Sequence Consistency Matching* (SCM) to retain a one-to-one mapping of *expert pass labels* and *imprecise pass labels* for evaluation. While this method enables the comparison of different amounts of passes from two data sources, we likely exclude some difficult examples from the evaluation. Thus, the obtained results in the testing procedure rather present an upper bound for the results in practice. In detail, SCM is performed by regarding sequences of active play and projecting the chronological order of two annotations within a sequence on to each other if and only the number of annotations within a sequence matches^46^.

We apply SCM using player identities from the *imprecise pass labels* and *expert pass labels*. However, if there exists a mismatch of pass labels within a sequence, it difficult to assign a *imprecise pass label* to its corresponding *expert pass label*. Thus, we decided to exclude the passes within these sequences for the evaluation. As a result, 1, 690 *consistent passes* were extracted for all four games of our dataset. Depending on the split in the cross-fold validation, the corresponding *consistent passes* of the test game are used for evaluation.

### Metrics

We evaluate our system with regard to two tasks: pass refinement precision and outlier detection. In the following, appropriate metrics for both tasks are presented.

*Frame-wise Accuracy of Pass Refinement:* To evaluate the temporal accuracy of refined pass predictions, we choose four different metrics. (1) The temporal distance (TD) describes the mean absolute error between the point in time of a *refined pass labels* to the corresponding *expert pass labels*. (2) In general, we aim to find the exact moment of a pass event. Thus, we propose to measure the fraction of exact annotations (EX) where the system outputs *refined pass labels* that occur at exactly the same frame as the corresponding *expert pass labels*. (3) To get an intuition about the amount of passes that are fairly well detected, we introduce a small error tolerance. This metric contains additional information to the mean absolute error which can generally be biased by single large values. We report the fraction of small errors (SE) for a maximum temporal distance of 0.48 s (12 frames) to the corresponding *expert pass label*. This maximum distance corresponds to a sampling frequency of 1 Hz which has been previously utilized for the analysis of passes^47^. (4) Despite we tried to ensure high-quality test samples with the sample assignment proposed in Section “Dataset”, in some cases assignments can still be invalid. To counteract their influence, we report the mean temporal distance of all pass refinements with small errors denoted as small temporal distance (STD). Please note, that for all previously presented metrics, we report the mean over the four splits.

*Pass Outlier Detection:* In order to assess the performance of the proposed outlier detection in Section “Pass event refinement and outlier detection”, we choose two additional metrics. We report the number of outliers (NOL) that describe the amount of neglected *refined pass labels* to evaluate the strictness of the filter. Admittedly, this metric alone does not describe the correctness of the detection. Therefore, we additionally report the outlier player-ball distance (OLPD) to evaluate the quality of the outlier detection.

The time of a pass event was carefully labeled at frame-level (Section “Introduction”), resulting in *expert pass labels*. This was achieved by regarding the distance of the ball to the foot of a player in the video data. Thus, it is expected that the player-ball distance in the position data is very small as well. However, we identified that in some cases the calculated distance of the passing player and the ball can be very large. We conclude that for these outliers (see Section “Pass event refinement and outlier detection”), the position data contains a significant amount of noise (see Section “Post-processing”). Therefore, we can evaluate the quality of the outlier detection by measuring this distance for all detected outliers. Moreover, this allows us to describe the total amount of removed noise in the outlier detection by a combination of the OLPD and NOL metrics. Please note that we accordingly report the aggregated NOL value and the mean OLPD value for the four splits.

### Parameter selection

In this section we aim to find the optimal parameters for different choices of segmentation, post-processing, and feature configurations for our proposed algorithm. We therefore perform a comparison of the synchronization performance for specific ranges of possible parameters, summarized in Table 2. Moreover, we also inspect the impact of the outlier detection for different tolerance thresholds by examining the position data quality of the detected outliers as well as of the remaining samples.

**Table 2:** Overview of varied parameters in the algorithm (see Table 1 for used features).

	Symbol	Evaluated values		
Window length [s]	*N*	1	1.8	2.6
Window shift [s]	*S*	–	0.04	–
Cutoff frequency of low-pass$\,[\text {Hz}]$	$f_c$	12.5	25	37.5
Feature representation	$\varvec{f}_{w}$	FR 1	FR 2	FR 3
Search interval [s]	–	–	$[-6, 0.8]$	–

Regarding the segmentation process we observe three different *window lengths* of $N = 1\,$s, $N = 1.8\,$s, and $N = 2.6\,$s. Here, we find that $N = 1\,$s is the minimum applicable *window length* which still contains the structural characteristics of a pass event (see Fig. 5, left). In contrast, we keep the minimum *window shift* of $S = 0.04\,$s (1 frame, regarding *sampling frequency* $f_s = 25$ Hz) constant as this relates to an online approach (see Section “Segmentation of player-ball distance and ball acceleration time-series”).

The examination of post-processing parameters is accomplished by regarding different low-pass filter cutoff frequencies of $f_c=12.5\,\text {Hz}$, $f_c=25\,\text {Hz}$, and $f_c=37.5\,\text {Hz}$ along a scenario without post-processing. For the influence of the feature space, we choose the three defined configurations FR 1–FR 3 (see Section “Feature extraction”). Finally, we select a constant *search interval* around the *imprecise pass labels* of $[-6\,\text {s}, 0.8\,\text {s}]$ since it contains over $97\,\%$ of the frame errors occurring in the used event data (see Fig. 3). Appropriately combining the different possible parameter choices yields 12 configurations for which we display the respectively obtained results in Table 3.

**Table 3:** Results in the introduced metrics from Section “Metrics” for different variants of *window length*, low-pass cutoff frequency, and feature representation.

Window length (s)	Cutoff frequency (Hz)	Features	TD (s)	STD (s)	EX (%)	SE (%)
$N=1$	–	FR 1	1.61	**0.09**	4.74	64.54
		FR 2	1.50	0.10	4.38	67.67
		FR 3	**1.40**	0.10	5.62	**71.05**
$N=1$	$f_c = 12.5$	FR 1	1.58	0.14	2.43	58.20
		FR 2	1.50	0.11	4.91	65.48
		FR 3	1.54	0.10	5.15	66.02
$N=1$	$f_c = 25$	FR 1*	1.51	0.12	5.74	68.92
		FR 2	1.44	0.10	6.22	69.03
		FR 3	1.44	0.10	6.57	69.03
$N=1$	$f_c = 37.5$	FR 1	1.55	0.10	5.86	67.97
		FR 2*	1.43	0.10	5.39	69.45
		FR 3*	**1.40**	0.10	**7.40**	70.34
$N=1.8$	–	FR 1	1.62	0.11	4.09	62.94
		FR 2	1.46	0.12	4.62	69.27
		FR 3	1.46	0.11	5.68	69.03
$N=1.8$	$f_c = 12.5$	FR 1	1.54	0.14	2.07	61.57
		FR 2	1.49	0.12	5.15	66.49
		FR 3	1.51	0.11	5.51	68.21
$N=1.8$	$f_c = 25$	FR 1	1.58	0.12	4.62	66.43
		FR 2	1.45	0.11	4.56	69.57
		FR 3	1.46	0.10	6.04	69.21
$N=1.8$	$f_c = 37.5$	FR 1	1.58	0.10	4.91	65.30
		FR 2	1.45	0.11	4.44	69.33
		FR 3	1.46	0.10	6.16	69.27
$N=2.6$	–	FR 1	1.61	0.13	3.49	60.69
		FR 2	1.48	0.13	3.85	65.13
		FR 3	1.51	0.11	4.26	66.55
$N=2.6$	$f_c = 12.5$	FR 1	1.57	0.14	2.13	58.56
		FR 2	1.47	0.13	4.44	66.31
		FR 3	1.48	0.12	5.39	68.98
$N=2.6$	$f_c = 25$	FR 1	1.58	0.12	4.32	62.88
		FR 2	1.45	0.13	4.14	67.91
		FR 3	1.50	0.10	5.51	67.38
$N=2.6$	$f_c = 37.5$	FR 1	1.59	0.11	3.55	60.51
		FR 2	1.47	0.13	4.68	67.61
		FR 3	1.50	0.10	4.97	67.44

Superior algorithm configurations (*) and results for each metric are printed in bold.

*General Findings:* The conducted experiments examine the general performance of the algorithm in the refinement of *imprecise pass labels* with respect to the influence of the selected parameters. Among the different examined algorithm configurations themselves, there persist relatively small differences concerning the presented metrics. This indicates the general robustness of the system in the investigated parameters.

*Segmentation:* Concerning the examined segmentation parameter, we find that the shortest applicable *window length* $N=1\,$s (see  “Parameter Selection” Section) performs best for all examined feature representations. Moreover, the results for all feature representations FR 1–FR 3 continually decrease when increasing the *window length* to $N=1.8\,$s and $N=2.6\,$s. This indicates that *window length* $N=1\,$s still contains the defining characteristics of pass events while larger *window lengths* add redundant information which has a negative effect on the pass event detection.

*Post-processing:* The application of a low-pass filter, in general, leads to positive effects in the examined metrics, however, only if the cutoff frequency $f_c$ is chosen accordingly. Here, the lowest value of $f_c=12.5\,\text {Hz}$ leads to inferior results compared to no postprocessing in all metrics and for all feature representations. Contrarily, the higher cutoff frequencies $f_c=25\,\text {Hz}$ and $f_c=37.5\,\text {Hz}$ have a largely positive effect on the obtained results while the achieved benefit varies along FR 1–FR 3. Reasons for this can be found in the higher degree of smoothing which simultaneously increases with the cutoff frequency. This illustrates that the post-processing is able to remove a certain amount of noise from the time-series which allows for a better generalization of pass events. Yet, for FR 3, the application of the low-pass filter with cutoff frequency $f_c = 37.5\,\text {Hz}$ leads to a small decrease of SE compared to no postprocessing. Consequently, the features of the more complex representation FC 3 are able to tolerate noise in the *time-series windows* to some degree. This indicates a dependence of parameters within the algorithm configuration and encourages a fine-tuning of the low-pass filter when applying the approach for a given configuration and dataset in practice.

*Comparison of Feature Representations:* From the obtained results we determine the optimal segmentation and post-processing parameters for each examined feature representation and denote them respectively FR 1*–FR 3*. Based on the lowest TD (and using EX as a tiebreaker) we determine the superior configurations of $N=1\,$s with $f_c =25\,$Hz for FR 1 and $N=1\,$s with $f_c =37.5\,$Hz for FR 2 and FR 3.

Among the superior configurations, the best TD, SE, and STD scores are obtained for FR 3* while the lowest scores are obtained for FR 1*. This correlates with the amount of complexity of the examined feature configurations which continuously increases from FR 1 to FR 3 (see Section “Feature extraction”).

Yet the superior algorithm configuration for the least complex feature representation FR 1 (consisting of just two features) achieves results close to the other feature representations in all metrics. Moreover, for EX, the superior algorithm configuration for FR 1* achieves a higher score than the superior configuration for FR 2*. This is likely caused by a larger influence of the individual features in the small-scale configuration FR 1. In detail, the feature describing the minimum of the *player-ball distance window* (see Section “Feature extraction”) generally provides a highly precise description of certain archetypal examples (i.e. direct passes). Consequently, a higher influence of this feature supports a frame-accurate annotation of passes in more cases which, however, does not apply for the general precision of the annotation. Thus, we recommend FR 1 for certain lightweight applications requiring a very low feature complexity i.e. due to memory or computation power limitations.

The more complex feature representation FR 2 comprises a total of four features, two for player-ball distance and two for ball acceleration. Compared to the results for FR 1*, FR 2* achieves an improvement in TD, STD, and SE. Consequent, we regard FR 2* as a good compromise of feature complexity and performance and recommend this configuration for applications seeking a low amount of performative features.

The most complex feature representation FR 3 comprises a total of 12 features, 10 regarding player-ball distance and two regarding ball acceleration. Since FR 3* achieves the best results in all metrics, we regard it as the optimal algorithm configuration and recommend it for the majority of applications.

**Figure 7:**
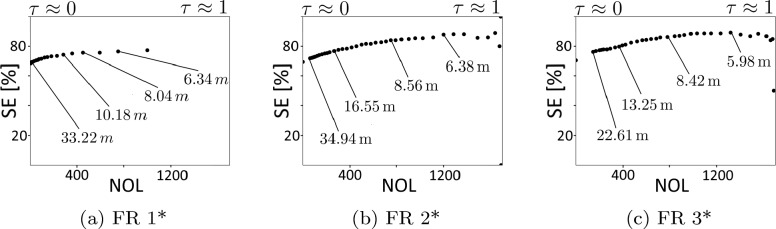
Results of the outlier detection in terms of small errors (SE) versus detected number of outliers (DNO) for *detection thresholds* $\tau$ in the range [0, 1]. At representative thresholds indicated are values of mean outlier player-ball distance (OLPD) that correlate with the impreciseness of the removed outliers.

**Figure 8:**
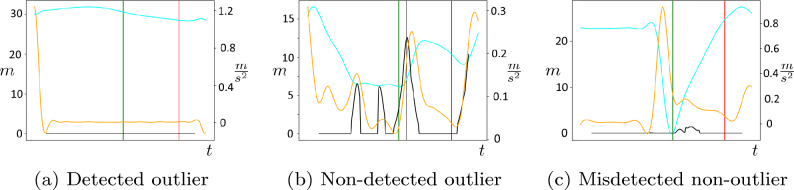
Qualitative Results for the outlier detection of the algorithm with displayed quantities similar to Fig. 10.

*Impact of the Outlier Detection:* The outlier detection was separately investigated for the superior algorithm configurations FR 1*–FR 3*. Examining different *detection thresholds* $\tau \in [0, 1]$, we individually compute the previously defined measures NOL and SE for each value. To assess the amount of noise in the position data for the detected outliers (see Section “Pass event refinement and outlier detection”), we additionally compute representative values of OLPD for a certain number of selected *detection thresholds*. The results for the outlier detection are displayed in Fig. 7. Additionally, we present qualitative examples in the outlier detection in Fig. 8.

For all examined configurations we observe an coincidental increase of NOL and SE along a decrease of OLPD with the *detection threshold* $\tau$. This illustrates the general capability of the *maximal pass event probability* in the *search interval*, $P(\varvec{f}^r_{w, \text {opt}})$ to serve as a conclusive confidence score regarding the certainty of the refinement decision.

Another indication for this is given by the OLPD metrics. In the entire dataset, the mean player-ball distance of all passes is given at 5.13 m. This value is exceeded by the OLPD values for the majority of *detection thresholds* for all feature configurations. Moreover, the OLPD values at *detection thresholds* $\tau \approx 0$, given at 32.22 m (FR 1*), 34.94 m (FR 2*), and, 22.61 m (FR 3*), largely surpass the mean. Therefore, we state that the algorithm is capable to detect localization errors in the position data at the *expert pass label*. Increasing the *detection threshold* causes a simultaneous decrease of OLPD among all examined feature configurations. This demonstrates a high correlation of the computed *pass event probabilities* and the amount of noise in the underlying position data.

In general, the results indicate that the outlier detection is a valuable extension to our proposed algorithm since it allows for highly precise fine-tuning between the quantity and quality of the obtained annotated data. However, the concrete decision on an optimal configuration and *detection threshold* highly depends on the requirements of a possible application. Accordingly, we recommend three configurations of the outlier detection and provide a comparison with the respective superior configurations without outlier detection in Table 4.

**Table 4:** Comparison of the recommended outlier detection with the respective superior algorithm configurations.

	NOL	OLPD (m)	TD (s)	STD (s)	EX (%)	SE (%)
FR 1*	–	–	1.51	0.12	5.74	68.92
FR 2*	–	–	1.43	0.10	5.39	69.45
FR 3*	–	–	1.40	0.10	7.40	70.34
FR 3*—OL$^\text {max}$	1114	6.48	1.23	0.11	9.04	88.52
FR 3*—OL$^\text {min}$	151	25.15	1.22	0.10	8.13	76.14
FR 3*—OL$^\text {opt}$	427	12.32	1.07	0.11	8.87	81.22

Regarding an application with high quality constraints for the utilized position data and a large available amount of data, we recommend OL$^\text {max}$ comprising FR 3* with $\tau = 0.675$. Here, we achieve a large improvement of SE, from $70.34\%$ without outlier detection to $88.52\%$ with outlier detection. The detected 1114 outliers have a mean player-ball distance of 6.48 m and the remaining 576 passes have a mean player-ball distance of 2.09 m which indicates the low amount of noise in the underlying position data.

In contrast, given a different application with strong limitations regarding the amount of available data we recommend performing the outlier detection with configuration OL$^\text {min}$ comprising FR 3* and $\tau = 0.025$. This configuration improves the initial SE of $70.34\%$ without outlier detection to $76.14\%$ while only 151 outliers with an OLPD of 25.15 m are detected.

As a compromise of the presented strategies, we propose the outlier detection OL$^\text {opt}$ comprising FR 3* and $\tau = 0.275$ since it detects 427 outliers with an OLPD of 12.32 m reliable and achieves a SE of $81.22\%$. However, please note that an application may also perform outlier detection as a preprocessing step with one feature configuration and perform the actual pass refinement with another.

### Comparison to baselines and state of the art

In the following section, we evaluate our proposed solution against meaningful baselines extracted from the original event data as well as a recent state-of-the-art approach for group activity detection^14^.  “Baselines” Section presents the different baselines in more detail. Finally, the results are presented and discussed in  “Comparison to baselines and state of the art” Section.

#### Baselines

Four baselines are examined in the scope of this experiment.

*Imprecise pass labels:* As a first baseline, we consider the *imprecise pass labels* from the event data. However, the comparison displayed in Fig. 3 of the *expert pass labels* to these *imprecise pass labels* reveals that the majority of passes are annotated after the corresponding *expert pass label*. This originates from the highly challenging real-time annotation process. The individual annotators usually capture a current event while simultaneously looking out for the following events in the match. Consequently, a rather reactive annotation scheme emerges as the anticipation of passes can only be established for certain rare cases. In contrast, during the annotation of the *expert pass labels* our annotator was able to pause and navigate the video for frame-accurate pass annotations while having no further restrictions regarding the duration of the process (see Section “Problem definition”).

*Statistical Baseline:* To counteract the issues of *imprecise pass labels* we suggest a statistical baseline that accounts for the average delay of the underlying annotation. Since typically different annotators are responsible to create event data for soccer matches, the error can depend on their individual characteristics and behaviors. Thus, we specifically compute a mean temporal distance between *imprecise pass labels* and *expert pass labels* for each match and half in our dataset. Based on this value, we perform a statistical refinement of the *imprecise pass labels* through an individual correction of each pass annotation by the respective mean temporal distance. According to Fig. 6 this intuitively relates to a shift of the origin of the x-axis to the mean of the respective match (and half, not displayed in the figure). The corrected annotations are then computed for each half and subsequently aggregated to define the *statistical baseline*.

*Classifier Baseline:* Regarding the evaluation of the used classifier in the pass event classification (see Section “Pass event detection”) we design a baseline that varies from the proposed solution only in the used classifier. Therefore, we adopt the previously presented pipeline (signal and feature extraction, pass event probability computation, pass event refinement refinement) as well as the training and test procedure (see  “Dataset” Section). Moreover, to obtain comparable results we decide on feature representation FR 3 (see Section “Feature extraction”) since it is the highest performing feature configuration. However, in contrast to our presented method, we compute the pass event probabilities using a simple logistic regression classifier^48^. Here, we increase the number of iterations such that the algorithm converges and balance the class weights to account for the highly imbalanced classification problem. Thus, a comparison with this baseline enables an isolated evaluation of the adapted SwiftEvent algorithm and its impact for the pass event refinement. Ultimately, we can also use this baseline to assess the performance of the proposed classifier in the outlier detection. Therefore, we present two additional configurations of the classifier baseline: one with minimal outlier detection (OL$^\text {min}$) that preserves the majority of data and one with optimal outlier detection (OL$^\text {opt}$) that achieves the best performance. The selection of these configurations is performed analogously to the procedure in  “Parameter Selection” Section.

*State-of-the-Art Baseline:* Due to the novelty of the task there exists no strictly similar pass synchronization concept in the related work which we can use to compare our results against. Moreover, datasets and approaches are typically not publicly available. Nevertheless, we still aim to compare our method with a (task-related) state-of-the-art model.

As described in Section “Introduction”, the detection of passes defined as the duration in which the ball travels between two players has been proposed^13^. However, due to the difference in our task, a comparison with this method is not possible. In a more general sense, a recent approach^14^ can be somewhat discussed with regard to the results reported in this paper. The authors consider the pass event detection as part of a general group activity detection framework. Yet, the observed problem differs from our approach in two main aspects. Firstly, the detection of events from the positional data is executed without additional information about the time, involved players, or the type of the event. Secondly, the algorithm is not limited to the detection of passes but furthermore detects shots and receptions in the match.

A prerequisite for this highly general approach is the initial problem of detecting an event in the first place. This problem is addressed by the implementation of a non-maximum suppression (NMS) procedure which only allows for a single prediction within a specified NMS window length. Preventing multiple detections within the window, this also addresses a problem regarding the assignment of detected passes, detailed in Section “Dataset”. Thus, the possibly induced positive bias can be partially accounted for by the NMS procedure, however, only if the utilized NMS window length (which is not reported) is chosen large enough.

#### Results and discussion

In the following section we report the results from the experiments comparing the optimal algorithm configurations to the introduced baselines. We evaluate the comparison to the event based baselines (in particular the *statistical baseline*) and to the *classifier baseline* independently and focus on the implications of both experiments. Finally, we assess the comparison of the proposed method to the state-of-the-art pass detection algorithm^14^. Here, we pay special attention to the fact that we adopted the reported metrics to compare the two algorithms, however, did not focus on optimizing them.

The results for the optimal configurations of our proposed solution as well as the baseline approaches are reported in Table 5. In addition to the proposed metrics in Section “Metrics”, we report results for ME (medium errors), Q50 (50 % quantile), and Q95 (95 % quantile)^14^ to allow a comparison to the state of the art. ME is given by the fraction of passes with a *refined pass label* with maximum temporal distance of 0.96 s (24 frames) to the corresponding *expert pass label*. Q50 and Q95 relate to temporal distances larger than or equal to 50 %, and 95 % of all errors, respectively.

**Figure 9:**
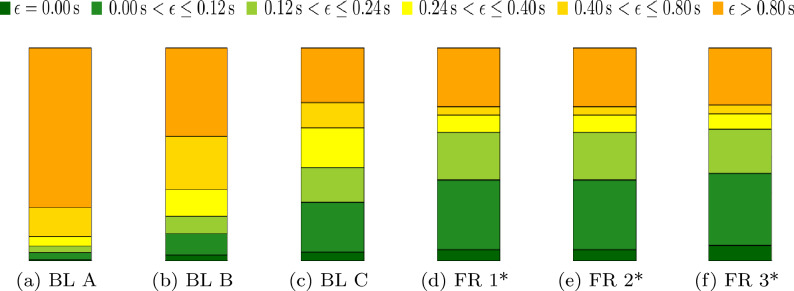
Visualization of the fraction of errors ($\epsilon$) within six different temporal ranges for different baselines (BL) and feature representations (FR) (**a**) *imprecise pass labels*, (**b**) *statistical baseline*, (**c**) *classifier baseline*, (**d**)–(**f**): the superior representations FR 1*–FR 3* of the synchronization algorithm.

Besides, we provide a visualization of error occurrences in Fig. 9 and indicate qualitative results for representative *imprecise pass labels* in Fig. 8.

**Figure 10:**
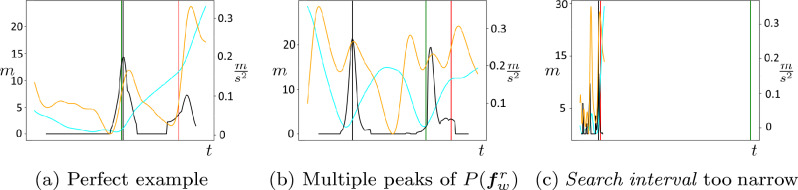
Qualitative Results of the algorithm for representative passes with displayed player-ball distance (cyan curve, in *m*) and ball acceleration (orange curve, in $\frac{m}{s^2}$) along the respective *expert pass label* (green line), the *imprecise pass label* (red line), and the *refined pass label* (black line, quantitative), which is computed from the algorithms *pass event probabilities* $P(\varvec{f}_w^r)$ (black curve) in the*search interval*.

**Table 5:** Results of the different baseline approaches *imprecise pass labels* (Baseline A), *statistical baseline* (Baseline B), *classifier baseline* (Baseline C), *classifier baseline* with outlier detection (BL C—OL$^\text {opt}$) and Baseline D (Sanford et al.^14^) as well as of different configurations of our proposed algorithm.

	Passes	TD (s)	STD (s)	EX (%)	SE (%)	ME (%)	Q50 (s)	Q95 (s)
Baseline A	1690	2.34	0.25	0.59	13.55	25.09	0.52	0.84
Baseline B	1690	1.70	0.24	3.02	49.88	58.64	0.40	0.84
Baseline C	1690	1.41	0.14	4.38	67.38	84.07	0.24	1.36
BL C - OL$^\text {opt}$	1312	1.30	0.15	5.16	75.29	87.03	0.20	0.44
FR 1*	1690	1.51	0.12	5.74	68.92	79.69	0.16	1.12
FR 2*	1690	1.43	0.10	5.39	69.45	81.65	0.16	1.28
FR 3*	1690	1.40	0.10	7.40	70.34	82.00	0.12	1.24
FR 3*—OL$^\text {min}$	1539	1.22	0.10	8.13	76.14	85.83	0.12	0.92
FR 3*—OL$^\text {opt}$	1263	1.07	0.11	8.87	81.22	87.40	0.12	0.28
**Baseline D**	**4560**	**–**	**–**	**–**	**–**	**87.00**	**0.20**	**0.48**

Bold: Please note, that results for Baseline D are reported for another dataset but are displayed here for comparison.

*Comparison to Baselines based on Event Data:* The results reveal that the *statistical baseline* (Baseline B) performs superior compared to the original *imprecise pass labels* (Baseline A). In turn, we find that the examined superior configurations of the proposed algorithm outperform the *statistical baseline* by a significant margin. In terms of TD, the results improve by up to $0.3\,$s compared to the baseline. The other metrics also improve: STD from 0.24 s up to 0.16 s, EX from $3.02\%$ up to $6.75\,\%$, and, SE from $13.55\%$ up to $70.83\,\%$. These values indicate a strong benefit of our proposed algorithm compared to (independent) statistical synchronization approaches.

*Comparison to the Classifier Baseline:* The *classifier baseline* (Baseline C) adopts the same workflow, features, and parameters as our the configuration FR 3* and is, thus, used to independently evaluate the role of our presented classifier in the framework. While the TD for the *classifier baseline* is able to outperform the results for FR 1* and FR 2*, the differences with respect to the feature configurations do not allow for a conclusive comparison of these results. More compelling is the comparison to FR 3* that uses the same features. Here, we find that our proposed algorithm performs superior to the *classifier baseline* for six out of seven metrics. Moreover, the slight advantage of the baseline in the ME metric is somewhat limited by the inferior results for Q50 and Q95.

This superiority of our proposed solution becomes more clear when investigating the baseline performance for applied outlier detection (BL C—OL$^\text {opt}$). While both algorithms have a similar number of detected outliers, the algorithm configurations with outlier detection outperform the baseline in all evaluation metrics. These results indicate that a more fine-grained confidence score is produced by our proposed classifier which is better suitable for the noise removal in position data.

An especially clear advantage of our proposed solution against the baseline is the performance in metrics that relate to the frame-wise accuracy of the detected passes (EX, SE). This is an important aspect for a broad number of applications such as visual action recognition tasks which often demand highly exact labels. Thus, we find that our adopted SwiftEvent^1^ classifier is a valuable component in the pass synchronization framework and that its high specificity regarding the classification task benefits the results.

*Comparison to the State of the Art:* In general, the state-of-the-art pass detection method^14^ is difficult to compare with our approach. Since the utilized data, as well as the source code, is not publicly available, we can not employ a common dataset. Therefore, for our proposed algorithm, we report results obtained by performing a four-fold cross-validation where we used three matches for training and one match testing. In contrast, the state-of-the-art machine learning algorithm^14^ performed training with 64 matches and testing with five matches. Moreover, the positional dataset from the 2018/2019 season is likely to contain significantly less noise than our dataset from 2014/2015.

Nevertheless, We adopt the evaluation metrics ME, Q50, and Q95 presented by the authors to enable a discussion of the performance of our proposed algorithm in relation to the state of the art^14^. However, please note that while we adopt these metrics from Sanford et al.^14^, we did not pursue an optimization in the parameter selection for our optimal algorithm configurations (see “Parameter Selection” Section). We present the reported measures and selected superior configurations of our approach in Table 5.

The obtained results reveal a general superiority of our proposed. While for the ME metric the results for FR 3* are inferior to the state of the art, the application of the outlier detection in FR 3—OL$^\text {opt}$ is able to outperform this ME result. Moreover, regarding the state-of-the-art quantile Q50 of $0.2\,$s, all examined variants of our algorithm achieve largely superior results. This is somewhat remarkable given that our data is expected to be significantly less precise. Regarding the state-of-the-art Q95 metric of $0.48\,$s, we find that while the standard configurations show inferior results (likely caused by the larger amount noise in the data), performing the outlier detection does also lead to superior results of 0.28 s.

Thus, we generally find that our proposed algorithm is capable to achieve a comparable amount of medium errors (ME) and simultaneously obtains a superior frame-exact accuracy, documented by the Q50 and Q95 values. Moreover, our probabilistic approach was trained on a significantly smaller and less recent position dataset. Finally, we emphasize that the state of the art did not describe or discuss any kind of localization errors in the position data^14^.

## Conclusions

Tracking technology has many applications in soccer and other sports domains. Yet, for more sophisticated analysis regarding team and player behaviors, companies^8^ provide (professional) clubs with additional match and player events such as shots, passes, etc. However, in this work, we have empirically shown that this event data is often temporally imprecise. To counteract this issue and allow for exact pass annotations this work has presented a novel framework for pass event refinement based on existing event data.

In a first step, features for player-ball distance and ball acceleration, obtained from the spatiotemporal position data, were extracted to construct a classifier for a general pass event detection that is based on SwiftEvent^1^. Subsequently, this classifier was employed to refine the existing pass events from the event data to fit the expert annotation. In this process, the classifier generates a respective confidence score which we further applied for the detection of localization errors, i.e. an inaccurate location of the ball, in the position data.

Experimental results have shown the superiority of the proposed solution in terms of the temporal accuracy of refined pass events compared to the annotations of existing event data and to another statistical baseline. This statistical baseline addresses the systematic error of delayed pass annotations in the original event data. Furthermore, we have shown that replacing our classifier by a logistic regression causes a significant decrease in performance, especially in the localization of errors. This promotes the choice of of our adaption of SwiftEvent^1^ as the optimal classifier in our framework.

An in-depth analysis of the various system parameters was conducted and has shown the robustness of the system as well as the efficiency of an outlier detection that removes unreliable positional data points. Parameter settings with various complexity were investigated and results have demonstrated that a lightweight solution can already improve the temporal accuracy of passes drastically. Due to the absence of a public test benchmark and common evaluation protocol, the overall performance of our proposed solution was discussed in relation to results from another more complex state-of-the-art approach for pass detection. Better performance was investigated for all evaluation metrics.

In the future, we assume a more significant improvement when utilizing data from video tracking systems or positioning systems (including three-dimensional ball position). Finally, we believe that, given a sufficiently large sample size of manual annotations, our algorithm may be modified to other events in the event data (e.g., shots and tacklings) to enable the synchronization with the position data.

### Supplementary Information

Supplementary Information.

## Data Availability

Due to licensing issues, the position and event data of the examined soccer matches cannot be made available. The dataset of *expert pass labels* are available from the corresponding author on reasonable request.

## References

[1] Gensler A, Sick B (2018). Performing event detection in time series with SwiftEvent: An algorithm with supervised learning of detection criteria. Pattern Anal. Appl..

[2] Deloitte Annual Review of Football Finance. https://www2.deloitte.com/uk/en/pages/sports-business-group/articles/annual-review-of-football-finance.html. Accessed 2021-04-1 (2020).

[3] Meyer T, Mack D, Donde K, Harzer O, Krutsch W, Rössler A, Kimpel J, Von Laer D, Gärtner BC (2021). Successful return to pro fessional men’s football (soccer) competition after the COVID-19 shut down: A cohort study in the German Bundesliga. Br. J. Sports Med..

[4] Rein R, Memmert D (2016). Big data and tactical analysis in elite soccer: Future challenges and opportunities for sports science. Springerplus.

[5] ChyronHego. https://www.chyronhego.com. Accessed 2021-01-21. (2021).

[6] Kinexon. https://www.kinexon.com. Accessed 2021-01-21 (2021).

[7] Bauer P, Anzer G (2021). A goal scoring probability model for shots based on synchronized positional and event data in football (soccer). Front. Sports Act. Living.

[8] OptaSports. https://www.optasports.com. Accessed 2021-01-19 (2021).

[9] Pappalardo L, Cintia P, Rossi A, Massucco E, Ferragina P, Pedreschi D, Giannotti F (2019). A public data set of spatio-temporal match events in soccer competitions. Sci. Data.

[10] Liu H, Hopkins W, Gómez AM, Molinuevo SJ (2013). Inter-operator reliability of live football match statistics from OPTA Sportsdata. Int. J. Perform. Anal. Sport.

[11] Chawla, S., Estephan, J., Gudmundsson, J. & Horton, M. Classifica tion of passes in football matches using spatiotemporal data. In *ACM Transactions on Spatial Algorithms and Systems, TSAS* **3**(2), 1–30 (2017).

[12] Power, P., Ruiz, H., Wei, X., & Lucey, P. Not all passes are created equal: Objectively measuring the risk and reward of passes in soccer from tracking data. In *ACM International Conference on Knowledge Discovery and Data Mining* 1605–1613. SIGKDD (2017)

[13] Sorano, D., Carrara, F., Cintia, P., Falchi, F. & Pappalardo, L. Automatic pass annotation from soccer VideoStreams based on object detection and LSTM. arXiv:2007.06475 (2020).

[14] Sanford, R., Gorji, S., Hafemann, L. G., Pourbabaee, B. & Javan, M. Group activity detection from trajectory and video data in soccer. In *Proceedings of the IEEE/CVF Conference on Computer Vision and Pattern Recognition Workshops* 898–899 (2020)

[15] Vidal-Codina F, Evans N, El Fakir B, Billingham J (2022). Automatic event detection in football using tracking data. Sports Eng..

[16] Bowerman BL, O’Connell RT (1979). Time Series and Forecasting.

[17] Chatfield C (2000). Time-Series Forecasting.

[18] De Gooijer JG, Hyndman RJ (2006). 25 years of time series forecast ing. Int. J. Forecast..

[19] Fu T-C (2011). A review on time series data mining. Eng. Appl. Artif. Intell..

[20] Guralnik, V., & Srivastava, J. Event detection from time series data. In *ACM International Conference on Knowledge Discovery and Data Mining, SIGKDD* 33–42 (1999)

[21] Yu M, Bambacus M, Cervone G, Clarke K, Duffy D, Huang Q, Li J, Li W, Li Z, Liu Q (2020). Spatiotemporal event detection: A review. Int. J. Digit. Earth.

[22] Aghabozorgi S, Shirkhorshidi AS, Wah TY (2015). Time-series clustering- a decade review. Inform. Syst..

[23] Liao TW (2005). Clustering of time series data-a survey. Pattern Recognit..

[24] Rani, S. & Sikka, G. Recent techniques of clustering of time series data: A survey. *Int. J. Comput. Appl.* **52**(15) (2012).

[25] Fawaz HI, Forestier G, Weber J, Idoumghar L, Muller P-A (2019). Deep learning for time series classification: A review. Data Min. Knowl. Disc..

[26] Geurts, P. Pattern extraction for time series classification. In *European Conference on Principles of Data Mining and Knowledge Discovery* 115–127. Springer (2001)

[27] Wei, L. & Keogh, E. Semi-supervised time series classification. In *ACM International Conference on Knowledge Discovery and Data Mining* 748–753. SIGKDD (2006)

[28] Makridakis S, Spiliotis E, Assimakopoulos V (2018). Statistical and machine learning forecasting methods: Concerns and ways forward. PLoS ONE.

[29] Li, Y., Lin, G., Lau, T. & Zeng, R. A review of changepoint detection models. arXiv:1908.07136 (2019).

[30] Lin J, Williamson S, Borne K, DeBarr D (2012). Pattern recognition in time series. Adv. Mach. Learn. Data Min. Astron..

[31] Bayat F, Moin MS, Bayat F (2014). Goal detection in soccer video: Role-based events detection approach. Int. J. Electr. Comput. Eng..

[32] Fakhar B, Kanan HR, Behrad A (2019). Event detection in soccer videos using unsupervised learning of Spatio-temporal features based on pooled spatial pyramid model. Multimed. Tools Appl..

[33] Jiang, H., Lu, Y. & Xue, J. Automatic soccer video event detection based on a deep neural network combined CNN and RNN. *IEEE 28th International Conference on Tools with Artificial Intelligence (ICTAI)* (San Jose, CA, USA), 490–494. 10.1109/ICTAI.2016.0081 (2016).

[34] Kapela, R., McGuinness, K., Swietlicka, A., & O’Connor, N. E. Real- time event detection in field sport videos. In: *Computer Vision in Sports* 293–316. Springer (2014)

[35] Liu, T., Lu, Y., Lei, X., Zhang, L., Wang, H., Huang, W. & Wang, Z. Soccer video event detection using 3D convolutional networks and shot boundary detection via deep feature distance. In *International Confer ence on Neural Information Processing, ICONIP* 440–449. Springer (2017).

[36] Saraogi, H., Sharma, R. A. & Kumar, V. Event recognition in broad cast soccer videos. *Indian Conference on Computer Vision, Graphics and Image Processing* 1–7 (2016).

[37] Tavassolipour M, Karimian M, Kasaei S (2014). Event detection and summarization in soccer videos using Bayesian network and copula. IEEE Trans. Circ. Syst. Vid..

[38] Yu, J., Lei, A. & Hu, Y. Soccer video event detection based on deep learning. In *International Conference on Multimedia Modeling* 377–389. Springer (2019)

[39] Zameni M, Fathy M, Sadri A (2010). A low cost algorithm for expected goal events detection in broadcast soccer video. Int. J. Digit. Content Technol. Appl..

[40] Zawbaa HM, El-Bendary N, Hassanien AE, Kim T-H (2012). Event detection based approach for soccer video summarization using machine learning. Int. J. Multimed. Ubiquitous Eng..

[41] Sanabria, M., Precioso, F. & Menguy, T. A deep architecture for mul timodal summarization of soccer games. *International Workshop on Multimedia Content Analysis in Sports* 16–24 (2019).

[42] Xu M, Maddage NC, Xu C, Kankanhalli M, Tian Q (2003). Creating audio keywords for event detection in soccer video. IEEE Int. Conf. Multimed..

[43] Van Oorschot, G., Van Erp, M. & Dijkshoorn, C. Automatic extrac tion of soccer game events from Twitter. In *DeRiVE@ ISWC* pp 21–30 (2012)

[44] Khan, A., Lazzerini, B., Calabrese, G., & Serafini, L. Soccer event detection. In *4th International Conference on Image Processing and Pattern Recognition* 119-129. IPPR. AIRCC Publishing Corporation (2018)

[45] Link D, Hoernig M (2017). Individual ball possession in soccer. PLoS ONE.

[46] Biermann, H., Theiner, J., Bassek, M., Raabe, D., Memmert, D. & Ewerth, R. A unified taxonomy and multimodal dataset for events in invasion games. In *Proceedings of the 4th International Workshop on Multimedia Content Analysis in Sports*. pp. 1–10 (2021)

[47] Rein R, Raabe D, Memmert D (2017). Which pass is better? Novel approaches to assess passing effectiveness in elite soccer. Hum. Mov. Sci..

[48] Kleinbaum DG, Dietz K, Gail M, Klein M, Klein M (2002). Logistic Regression.

