# Supplementary material for: Synchronization of passes in event and spatiotemporal soccer data
Source: Sci Rep. 2023 Sep 23;13:15878. doi: 10.1038/s41598-023-39616-2 (PMC10518005; doi:10.1038/s41598-023-39616-2)
Supplement: Supplementary file 1 — Supplementary Information. [file 41598_2023_39616_MOESM1_ESM.pdf]

Appendix

Formulation of Least-Squares Approximation Problems

We first make some remarks on the solution of linear least-squares problems before we present an approximation of a time-series with orthogonal polynomials.

Assume we are given a time-series consisting of real-valued observations y_i at points in time x_i with $i = 0, 1, \ldots, N$. We further assume that these points are equidistant in time. A common practice in many applications in statistics and other applied sciences is to construct a curve that is considered to be the "best fit" for the data, in some sense, that can be used to represent the set of data. One of the basic methods of approximations is the *least-squares approach.*

We wish to approximate the time-series with a polynomial that minimize the least-squares error. The polynomial $p_{\boldsymbol{w}}(x) : \mathbb{R} \to \mathbb{R}$ fitted to the observations y_i at (equidistant) points in time x_i can be represented by a linear combination of certain (linear independent) basis polynomials p_k:

$$p_{\boldsymbol{w}}(x) = w_0 p_0(x) + w_1 p_1(x) + \cdots + w_M p_M(x) = \sum_{k=0}^{M} w_k p_k(x) \tag{1}$$

with $M << N$, where $\boldsymbol{w} = [w_0, \ldots, w_M]^T \in \mathbb{R}^{M+1}$ is the (unknown) coefficient vector. If we write the values of the M basis polynomials $p_k(x)$, $k = 0, \ldots, M$, for the $N + 1$ data points $x_0, x_1, \ldots, x_N$ into a matrix

$$\boldsymbol{P} = \begin{bmatrix} p_0(x_0) & \cdots & p_M(x_0) \\ \vdots & \ddots & \vdots \\ p_0(x_N) & \cdots & p_M(x_N) \end{bmatrix} \in \mathbb{R}^{(N+1)\times(M+1)} \tag{2}$$

and define a vector $\boldsymbol{y} = [y_0, y_1, \ldots, y_N]^T \in \mathbb{R}^{N+1}$, then the linear least-squares minimization problem that we want to solve is

$$\min_{\boldsymbol{w}} \|\boldsymbol{P}\boldsymbol{w} - \boldsymbol{y}\|, \tag{3}$$

with $\|\cdot\|$ being the Euclidean norm. Minimizing (3) with respect to the coefficients leads to

$$\tilde{\boldsymbol{w}} = (\boldsymbol{P}^T\boldsymbol{P})^{-1}\boldsymbol{P}^T\boldsymbol{y} = \boldsymbol{P}^{+}\boldsymbol{y}, \tag{4}$$

where $\boldsymbol{P}^{+} = (\boldsymbol{P}^T\boldsymbol{P})^{-1}\boldsymbol{P}^T$ is the *pseudo-inverse* of $\boldsymbol{P}$. Thus, the approximation polynomial (1) is reduced by utilizing equation (4) [3, 5]. The (average) *squared error* σ^2 is referred to the residual vector $\boldsymbol{r} = \|\boldsymbol{P}\tilde{\boldsymbol{w}} - \boldsymbol{y}\|^2$ divided by the number of observed samples [2, 3], $N + 1$, i.e.

$$\sigma^2 = \frac{1}{N+1}\|\boldsymbol{P}\tilde{\boldsymbol{w}} - \boldsymbol{y}\|^2 = \frac{1}{N+1}(\boldsymbol{y}^T\boldsymbol{y} - \tilde{\boldsymbol{w}}^T\boldsymbol{P}^T\boldsymbol{P}\tilde{\boldsymbol{w}}). \tag{5}$$

The representation of the approximating polynomial $p_{\boldsymbol{w}}(x)$ in (1) may be a linear combination of the first $M+1$ monomials $1, x, \ldots, x^M$. This choice of the basis polynomials may be not numerically effective since the system matrix $\boldsymbol{P}$ is often ill-conditioned as the degree M increases. The main issue stems from the fact that the monomials as basis polynomials look increasingly similar as we take higher and higher powers. In addition, we do not know in advance which degree will give a satisfactory solution. Thus, if the degree of the polynomial is changed then using the monomials as basis polynomials the previous coefficients have to be recomputed. One way to avoid these disadvantages is by using basis polynomials which allows for the raising of the degree of the polynomial, while the coefficients already computed retain their value. By transforming the monomials into a special type polynomials having the property of being *orthogonal* we can attain our goal.

Now, assume that the selected basis polynomials in (1) are pairwise *orthogonal*, i.e $\sum_{n=0}^{N} q_i(x_n)q_j(x_n) = 0$ for any two q_i and q_j with $i \neq j$. Then, the matrix $\boldsymbol{P}$ in (2) has orthogonal columns and, thus $\boldsymbol{P}^T\boldsymbol{P}$ is now a diagonal matrix with the squared norms of the basis polynomials $q_k(x)$ as diagonal elements. If the elements in the diagonal are non-zero, then we get

$$w_k = \frac{1}{\|q_k\|^2} \sum_{n=0}^{N} y_n q_k(x_n). \tag{6}$$

That is, the least-squares solution can be determined from the observations $y_0, y_1, \ldots, y_N$ in a time window and, thus, each coefficient w_k can be found independently from the others.

With (5) and (6) the (average) squared error σ^2 is now [2, 3]

$$\sigma^2 = \frac{1}{N+1}\left(\sum_{i=0}^{N} y_i^2 - \sum_{n=0}^{M} w_n^2 \|q_n\|^2\right). \tag{7}$$

The error (7) may be used as an additional feature [4].

There are a variety of ways to generate orthogonal polynomials [1, 3, 7]. In our work, the polynomials $q_0, q_1, \ldots, q_M$ are built from the monomials $1, x, \ldots, x^M$ by the *Gram-Schmidt-Orthogonalization Process* [6, 8].

List of Examined Features

A list of all features which were extracted for the *time-series windows* and examined in the experiments is given in Table 1.

Table 1 Overview of examined features

Feature	Description	Size
Polynomial weights	Weights of the orthogonal polynomials when performing an approximation up to degree M	M
Polynomial error	Error of a polynomial approximation of degree M to the *time-series window*	1
Polynomial spline weights	Weights of the orthogonal polynomials when performing an approximation up to degree M for G splines of the *time-series window*	MG
Coarsening values	Aggregation of values for C parts of the *time-series window*	C
Derivative	The D order derivative of the *time-series window*	N
Mean	Mean of the *time-series window*	1
Minimum	Local minimum of the *time-series window*	1
Maximum	Local maximum of the *time-series window*	1
Minimum ratio	Ratio of the two smallest local minima in the *time-series window*	1
Maximum ratio	Ratio of the two smallest local maxima in the *time-series window*	1
Minimum position	Position of the local minimum of the *time-series window*	1
Maximum position	Position of the local maximum of the *time-series window*	1
Curvature mean	Mean of the *time-series window* curvature values	1
Curvature minimum	Local minimum of the *time-series window* curvature	1
Curvature maximum	Local maximum of the *time-series window* curvature	1
Curvature minimum ratio	Ratio of the two smallest local minima in the *time-series window* curvature	1
Curvature maximum ratio	Ratio of the two smallest local maxima in the *time-series window* curvature	1
Curvature minimum position	Position of the local minimum of the *time-series window* curvature values	1
Curvature maximum position	Position of the local maximum of the *time-series window* curvature values	1
Window center value	Value in the *time-series window center*	1
Window center curvature	Curvature value in the *window center*	1
